# OpenOrbitalOptimizer—A Reusable Open Source Library for Self-Consistent Field Calculations

**DOI:** 10.1021/acs.jpca.5c02110

**Published:** 2025-06-11

**Authors:** Susi Lehtola, Lori A. Burns

**Affiliations:** † Department of Chemistry, 111628 University of Helsinki, P.O. Box 55, FI-00014 University of Helsinki, Finland; ‡ Center for Computational Molecular Science and Technology, School of Chemistry and Biochemistry, 1372 Georgia Institute of Technology, Atlanta, Georgia 30332, United States

## Abstract

According to the modern paradigms of software engineering, standard tasks are best accomplished by reusable open source libraries. We describe OpenOrbitalOptimizer: a reusable open source C++ library for the iterative solution of coupled self-consistent field (SCF) equations ***F***_*p*_^σ^({***C***_*p*_^σ^}) ***C***_*p*_^σ^ = ***C***_*p*_^σ^ ***E***_*p*_^σ^ for an arbitrary number of particle types *p* and symmetries. Although OpenOrbitalOptimizer is a new project, it already implements standard algorithms for solving SCF equations: Pulay’s direct inversion in the iterative subspace (DIIS), energy DIIS (EDIIS), augmented DIIS (ADIIS), and the optimal damping algorithm (ODA). The library was designed as an easy way to introduce state-of-the-art convergence accelerators in a number of legacy programs. It is easy to interface with various programs, as it only requires a function to evaluate the total energy *E* and Fock matrices {***F***_*p*_^σ^} for a given set of orbitals {***C***_*p*_^σ^}. The only assumption behind the library is that one is able to easily store Fock and orbital matrices in memory, and to diagonalize the Fock matrices in full, which is the case in the overwhelming majority of quantum chemistry applications. We exemplify the library with nuclear-electronic orbital (NEO) calculations of protonated water clusters with Gaussian-type orbital basis sets. We find that a minimal-basis protonic guess works well, and that the stepwise SCF algorithm requires less computational time than the simultaneous SCF algorithm.

## Graphical abstract

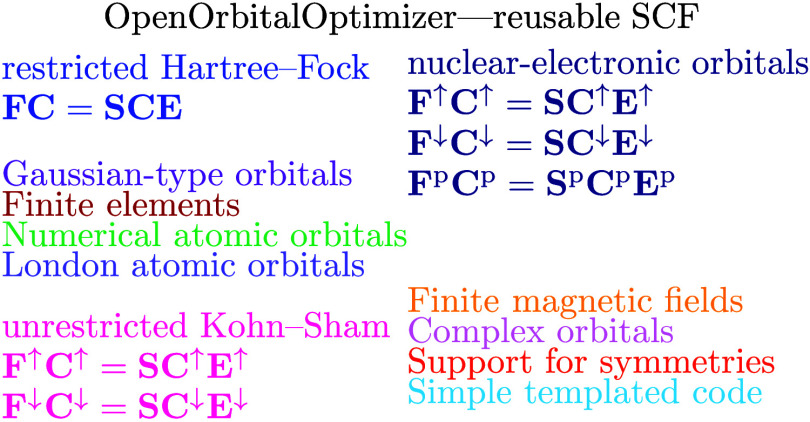

## 1 Introduction

Electronic structure calculations have become widely used in academia and industry to explain and predict the properties of materials, and the various steps involved in chemical reactions, for example. A typical electronic structure calculation involves finding the electronic one-particle states also known as orbitals. These orbitals are usually found by minimizing the Hartree–Fock or Kohn–Sham density functional^1,2^ for the total energy *E*[{ψ_*iσ*_}],^3^ which depends on the occupied set of the orbitals ψ_*iσ*_ of spin σ. Depending on the type of the system, the orbitals ψ_*iσ*_ are called atomic, molecular, or crystalline orbitals for atoms, molecules, and periodic systems, respectively.

To allow for a computational solution, the orbitals must be discretized in some form. The typical choice in quantum chemistry is the linear combination of atomic orbitals (LCAO) method, especially when combined with the use of Gaussian-type orbital (GTO) basis functions.^4^ In LCAO, the orbitals are written in the basis set χ_μ_(***r***) as

$$\psi_{i\sigma }\left(r\right)=\underset{\alpha }{\sum }C_{\mu i}^{\sigma }\;\chi_{\mu }\left(r\right)$$ 1

and the energy is now minimized with respect to the orbital expansion coefficients ***C*** ^σ^. However, since eq 1 obviously contains no assumption on the form of the basis functions, it can also be used in combination with other types of basis sets, such as finite elements, for example.

The variation of the total energy functional with respect to the orbital coefficients leads to the Roothaan-type equation^3,5–7^

2 $$F^{\sigma }\left(\{P^{\sigma }\}\right)C^{\sigma }={\mathrm{SC}}^{\sigma }E^{\sigma }$$

where *F*_*μν*_^σ^ = ∂*E*/∂*P*_*μν*_^σ^ is the Fock matrix, *S*_*μν*_ = ⟨μ|ν⟩ is the overlap matrix of the basis set, ***E*** ^σ^ is a diagonal matrix holding the orbital energies, and

3 $$P_{\mu \nu }^{\sigma }=\sum_{i}f_{i\sigma }C_{\mu i}^{\sigma }{\left(C_{\nu i}^{\sigma }\right)}^{†}$$

is the density matrix where *f*_*iσ*_ are the orbital occupation numbers. Any solution to eq 2 is a critical point of the total energy; typically, one hopes that the solution is a local (if not the global) minimum, but the self-consistent solution of eq 2 can also converge to a saddle point.

The central task now becomes to find such a set of orbitals ***C*** ^σ^ that generates Fock matrices ***F*** ^σ^ that yield the same ***C*** ^σ^ upon the solution of eq 2; this is known as the self-consistent field (SCF) problem.^3^ The SCF method consists of an iterative procedure: starting from some initial guess for the orbitals ***C*** ^σ^ (which can be also obtained from a guess for the Fock matrices ***F*** ^σ^, or density matrices ***P*** ^σ^ that will be used to build such guess Fock matrices),^8–11^ one computes a new Fock matrix, which in turn determines a new set of orbitals.

The Roothaan equation is typically solved by re-expressing the unknown orbitals in an orthonormal basis set^3^

4 $$C^{\sigma }=X{\mathrm{C̃}}^{\sigma }$$

and left-projecting eq 2 with ***X*** ^T^, which reduces the problem to a normal eigenvalue equation

5 $${\mathrm{F̃}}^{\sigma }\left(\{P^{\sigma }\}\right){\mathrm{C̃}}^{\sigma }={\mathrm{C̃}}^{\sigma }E^{\sigma }$$

where

6 $$\mathrm{F̃}=X^{T}\mathrm{FX}$$

in the case that ***X*** is chosen such that ***X*** ^T^ ***SX*** = **1**. Such matrices ***X*** can be formed with symmetric orthogonalization,^12^ or canonical orthogonalization when the basis set has linear dependencies.^13^ If the basis set contains many linear dependencies, a smaller basis set that spans the same space can be first found with a pivoted Cholesky decomposition before carrying out the conventional orthonormalization procedure.^14,15^

Eqs 4 to 6 define the Roothaan update, enabling one to determine new orbitals from the obtained Fock matrix. However, the central issue in SCF calculations is that this procedure of iterative Fock builds and diagonalizations is insufficient for all but the simplest of systems.

To begin, the Roothaan procedure for Hartree–Fock can be shown to either converge to a critical point of the Hartree–Fock functional (i.e., a solution of eq 2), or to converge asymptotically to a pair of density matrices, each iteration alternating between the two in an effect commonly known as *charge sloshing* (see Cancès and Le Bris^16,17^ for the proof). For this reason, SCF convergence has typically to be stabilized in the early iterations in order to damp down this sloshing.

Another issue is that even in the case when the Roothaan iterations converge onto a critical point of the functional, the convergence can be too slow for practical applications. Methods to extrapolate the Roothaan updates are therefore desirable.

Various schemes have been developed to address these two issues in SCF calculations. As the literature is very broad, we will only present the most important aspects of the literature here, postponing the more thorough discussion to a dedicated review article.

While convergence acceleration near the converged solution has long been typically handled with the direct inversion in the iterative subspace^18^ (DIIS) approach of Pulay,^19^ determining a wave function that is sufficiently close to the solution for DIIS to be applicable is traditionally the harder problem, especially when accurate initial guesses^8–11^ are not available in the employed electronic structure program: when a poor quality guess such as the one-electron guess obtained by omitting all electronic interactions from the Fock matrix is used, the guess Fock matrix and the orbitals are far from their optimal values, and significant changes in the orbitals and their occupations are typically observed in the first iterations.

The traditional approach to stabilizing convergence is to damp the density matrix update; an optimal damping algorithm has been suggested by Cancès and Le Bris.^20^ Level shifting^21^ is another commonly used alternative, and it has been shown^16,17^ that a sufficiently large level shift always leads to progress toward the minimum; however, the rate of convergence also becomes the slower the larger the shift is.

Newer approaches to allow robust SCF convergence include using physically motivated proxy energy functionals in combination with a DIIS type approach,^22,23^ as well as various procedures based on the direct minimization of the energy with respect to orbital rotations.^24–34^

Still, a major problem in practical electronic structure calculations today is the lack of state-of-the-art SCF algorithms in many program packages, which poses severe issues for ease-of-use and computational efficiency of these packages. While state-of-the-art SCF algorithms can often quickly and reliably converge even challenging electronic structures, such as those found in transition metal complexes, traditional SCF algorithms may require hundreds more SCF iterations to reach convergence, or fail to converge at all, necessitating user action.

A further complication is that many packages lack good initial guesses, which makes the orbital optimizations much more challenging than when a qualitatively correct initial guess is employed. Atomic guesses are typically already close to the DIIS convergence region, but sadly, many programs focusing on post-Hartree–Fock calculations are limited to the guess corresponding to the one-electron (core) Hamiltonian.

The central issue behind these limitations is that initial guesses and orbital optimizers are commonly deeply tied to the electronic structure package. In fact, the base layer of any electronic structure program habitually consists of the integrals and the SCF implementation. This tight embedding arises from the traditional monolithic development model of electronic structure software, which has many downsides, as discussed recently by Oliveira et al.^35^ and Lehtola,^36^ for example: it leads to an approach that is both laborious and error prone, as new algorithms always need to be reimplemented from scratch in every package. The considerable duplication of work makes it hard to propagate improved algorithms to various program packages.

The lack of standard implementations also causes issues with the lack of reproducibility between program packages. As discussed by Hinsen^37^ and Lehtola and Karttunen,^38^ software implementations of algorithms tend to be much more complicated than their journal descriptions, containing many aspects and features which are never discussed in the article. These aspects are brought to the open only if the implementation is open source, in that it is freely available at no cost for whatever use by whomever.^38^ As the “war over supercooled water”^39^ discussed by Lehtola and Karttunen^38^ has showed in practice, falsifiability of the results—an elementary requirement of the scientific method—is only possible if the source code used to obtain the results is freely and openly available.

Orbital optimization and SCF algorithms are extremely good examples of algorithms whose implementation is complicated enough that many aspects of the actual implementations cannot be found in journal articles. Although the field remains active in the literature,^33,34,40–43^ it is often difficult to compare to the state of the art as many implementations are in programs that are not openly available.

However, reusable open source software offers a solution.^36^ Following the discussion in ref 36, reusable libraries can be seen as the pinnacle of scientific software development: supplying a general solution to an important scientific problem (always within some limited scope) shows that one has both identified and solved the problem. Although the state of the art in the solution techniques can change as new methods are introduced in the literature, reusable open source libraries best satisfy the requirements of the art of the trade (good scientific practices), as open source library implementations can be kept more easily up to date than duplicate implementations in individual program packages. The open source aspect is important here (see Hocquet and Wieber,^44^ Lehtola and Karttunen^38^ and Lehtola^36^ for discussion): only when the implementation is freely accessible to *all potential users* and it can be studied and changed at source code level without having to sign a nondisclosure agreement has the problem been actually solved; there is a close analogy here to the push toward open access publishing of scientific articles, which has already become a generally accepted goal in many countries around the world.

In this work, we describe OpenOrbitalOptimizer:^45^ a reusable open source C++ library for orbital optimization and SCF calculations. As far as we are aware, OpenOrbitalOptimizer is the first of its kind: a flexible SCF library designed to be interoperable with a large number of electronic structure packages, and compatible with a wide variety of electronic structure methods. Previous efforts at reusable libraries for SCF calculations appear to be limited to the diagonalization step: for instance, the eigenvalue solvers for petascale applications (ELPA) library,^46^ the orbital minimization method (OMM) library,^47^ as well as the electronic structure solver infrastructure^48,49^ (ELSI) all target the solution of a single step of eq 2 (possibly combined with eq 3), instead of the self-consistent solution.

OpenOrbitalOptimizer grew out of a realization that the current situation is untenable: for instance, the orbital optimizers in ERKALE,^50^ HelFEM,^51–53^ Psi4,^54^ PySCF,^55^ and OpenMolcas^56^ are all disparate, even though they all use similar algorithms with similar implementations. The implementation of novel algorithms separately in every program package implies significant effort—and potential for bugs—and the main goal of this work is to demonstrate that orbital optimization *can* be outsourced to a dedicated reusable library.

The specific reason to develop OpenOrbitalOptimizer now is our push toward new quantum chemistry software employing numerical atomic orbitals (NAO),^4,52,53,57–59^ which will be based on reusable library implementations to the maximal degree possible: rather than developing a traditional monolithic program, we realized that a focus on reusable components can have a much more wide ranging impact,^36^ since the new reusable components can be useful also in existing program packages.

A reusable SCF implementation in a modular open source library allows the use of state-of-the-art orbital optimizers across various electronic structure packages. Importantly, the orbital optimization algorithm itself is typically not a bottleneck of the calculation; instead, most of the time in calculations is usually spent on evaluating the total energy and the Fock matrix, i.e., orbital gradient for a given set of orbitals. A smarter choice for the orbital optimizer can significantly reduce the number of steps needed to converge onto a (local) minimum, thus saving (potentially a considerable amount of) computing resources.

In addition, a reusable SCF implementation can also be easily interfaced to legacy programs. Even though modifications to the deep base layers of the existing SCF frameworks of legacy programs tend to be unattractive, a reusable SCF implementation with state-of-the-art solvers can still be used to predetermine converged orbitals, which can then be fed into the pre-existing SCF code as an initial guess. The legacy SCF implementation will simply reach convergence with a single step, bypassing the need to make laborious modifications to or refactors of the legacy code.

Furthermore, a smarter SCF implementation can make calculations considerably easier to run, which is especially important in the current age of big data where artificial intelligence applications may require running millions of SCF calculations to supply training data to machine learning models. Abstraction of SCF algorithms across various electronic structure programs can eliminate the need to set up convoluted workflows in case a given SCF algorithm fails to work for some data points. The traditional solutions of changing the initial guess, adjusting the density damping factor or level shift *etc.* simply do not scale to massive data applications.

Finally, a reusable SCF implementation can also greatly simplify the introduction of improved initial guesses in both novel and legacy programs. The implementation of the atomic density guess employing fractional occupations, for example, requires extensive changes to the SCF logic;^53^ however, the guess is easy to implement if a flexible SCF implementation is available.

A key motivation for the development of OpenOrbitalOptimizer are the recent developments in quantum chemistry for calculations in strong magnetic fields, where complex-valued orbital solvers become necessary in general,^60,61^ as well as multicomponent calculations within the nuclear-electronic orbital (NEO) method^62–64^ that require the simultaneous solution of Schrödinger equations for electrons and protons, or other quantum particles. Although the equations can be solved by alternating between the solution of the electronic and protonic equations, since the problems are strongly coupled, it has been shown that the simultaneous solution converges faster.^41,65^ A key design criterion of OpenOrbitalOptimizer was thus to also enable facile implementation of complex-valued SCF procedures as well as NEO-type models, which feature other types of quantum particles in addition to electrons.

The organization of the manuscript is the following. Next, we will present the theory of SCF calculations in section 2. Then, we will discuss the basic design choices made in OpenOrbitalOptimizer in section 3, and present the computational details of the calculations performed in this work in section 4. We will discuss the results demonstrating the novel implementation in section 5, and finish with a summary and conclusions in section 6.

## 2 Methods

We start out by simplifying the notation. As even in the case of a nonorthonormal basis the solution of eq 5 is carried out as a standard eigenvalue problem through eqs 4 and 6, we write eq 5 in the form

7 $$F^{\sigma }\left(\{C^{\sigma }\}\right)C^{\sigma }=E^{\sigma }C^{\sigma }$$

where it is now implied that the quantities are defined in the orthonormal basis. We underline that this is not a restriction, since in practice SCF convergence acceleration has to be carried out in the orthonormal basis, anyway.^18,19^ OpenOrbitalOptimizer works exclusively in a basis of orthonormal orbitals, which has the benefit of making the code simpler.

Next, we can make a number of observations that allow us to generalize the implementation as well as make it more powerful and efficient. First, we realize that the spin dependence in the Roothaan equations can be generalized to the case of an arbitrary number of types of particles *p*

8 $$F_{p}\left(\{C_{p}\}\right)C_{p}=E_{p}C_{p}$$

The spin-restricted case features a single type of particle (electrons), whereas spin-polarized calculations feature two types of particles: one for the spin-up electrons, and another for the spin-down electrons. The huge benefit of this generalization is that also multicomponent methods such as NEO fit seamlessly in the library. Spin-restricted NEO has two types of particles: the spin-restricted electrons, and the quantum protons, which are usually assumed to be in a high-spin state.^62–64^ Spin-unrestricted NEO again splits the electrons into a further two types (spin-up and spin-down electrons), so there are three types of particles in total. The implementation supports an arbitrary number of types of particles, and a single loop over the particle types suffices to solve all the Roothaan equations.

Second, if symmetry is employed, the Fock matrices have a block-diagonal structure

$$F=\left(\begin{array}{lll} F_{\mathrm{symm}\;1} & 0 & 0\\ 0 & \ddots & 0\\ 0 & 0 & F_{\mathrm{symm}\;n} \end{array}\right)$$

the off-diagonal blocks vanishing as they would mix symmetries. This means that the solution of the Roothaan equations for each type of particle can be split into its symmetry blocks, each of which yield corresponding orbital coefficients and orbital energies. The most typical symmetry employed in quantum chemistry packages is Abelian symmetry, which results in the maximum of 8 symmetry blocks corresponding to the *A*_*g*_, *B*_1*g*_, *B*_2*g*_, *B*_3*g*_, *A*_*u*_, *B*_1*u*_, *B*_2*u*_, and *B*_3*u*_ blocks in the highest *D*_2*h*_ symmetry; Abelian symmetries can also be found at finite magnetic fields.^66^ Other examples include atomic symmetry (*s*, *p*, *d*, *f*, ... orbitals) and linear symmetry (σ, π, δ, φ, ... orbitals), which are likewise useful in relevant contexts.^4,51–53,57^ The implementation supports an arbitrary number of symmetry blocks, which are defined by the calling program.

Each symmetry block for a given particle type typically has a different size. Because all operations are independent for each type of particle and symmetry block, the SCF problem is unambiguously defined in terms of the individual symmetry blocks, each of which correspond to some type of particle. The symmetry blocks thus form the elementary data structure in the implementation.

Third, to account for various handlings of the spin, symmetry, and differences in the ways occupations are handled for the various types of particles (e.g., in the NEO method), a maximal occupation *f* ^max^ must be defined separately for the orbitals in each block. For instance, a spin-restricted calculation without symmetry (*C*_1_ symmetry) fits 2 electrons per spatial orbital, while a spin-unrestricted calculation fits 1 electron per spin–orbital. When occupations are averaged,^53^ an atomic orbital with angular momentum *l* can fit 2*l*+1 electrons in a spin-unrestricted calculation, and again double the amount in a spin-restricted calculation. The abstraction of the maximum occupation allows updates to occupations to be easily handled with a single routine. Defining the total number of particles of each type, and the maximal orbital occupation in each symmetry block, the Aufbau configuration can be formed by looping over all particle types, and then distributing the specified number of particles across the orbitals of all symmetry blocks for that type of particle in increasing energy.

Fourth, although the mainstream of quantum chemistry is carried out with real-valued orbitals, complex-valued orbitals are necessary in some contexts. As already mentioned in the Introduction, orbitals in general become complex in the presence of finite magnetic fields,^60,61^ and one of the main motivations in this work is to introduce reusable software infrastructure that can also be used within this context. Complex wave functions sometimes also arise in molecular problems, as discussed by Small et al.^67^

Calculations with periodic boundary conditions are another stereotypical example for the need of complex arithmetic, the problem here being to solve Roothaan equations at every ***k***-point in the first Brillouin zone^68^

9 $$F^{\sigma }\left(k\right)C^{\sigma }\left(k\right)=S^{\sigma }\left(k\right)C^{\sigma }\left(k\right)E^{\sigma }\left(k\right)$$

where the Fock and overlap matrices are in general complex for ***k*** ≠ **0**; note here that ***k*** and σ label symmetry blocks. Since SCF acceleration in periodic calculations follows closely the molecular approach,^69^ and eq 9 is still of the same form as eq 2, the library should also support periodic calculations without changes. If necessary, the code is straightforward to amend to allow **k**-point weighting in the convergence acceleration.^69,70^

Fully relativistic four-component calculations offer another example of the need of complex-valued SCF, relativistic effects being famously important for the chemistry of heavy elements.^71,72^ The main complication in four-component calculations is the Dirac sea of negative-energy solutions, which correspond to positronic solutions.^73^ One should only populate the positive-energy orbitals that correspond to electrons, and this is achieved by skipping over the negative-energy solutions when determining the occupations of the four-component spinors.^73^ The number of negative-energy solutions is given by the number of (linearly independent) small-component basis functions in the calculation, which is thus known beforehand. Although this feature does not exist at present in OpenOrbitalOptimizer, the required change is thus trivial.

Note that relativistic effects are often described within approaches that do not feature the Dirac sea, the negative energy states being projected out by design. Popular approaches in present-day quantum chemistry include the use of effective core potentials,^74^ and the use of the exact two-component (X2C) method.^75–78^ In contrast to the four-component calculations discussed in the previous paragraph, these methods do not cause any extra complications, as the SCF is carried out for these methods exactly in the same way as in the nonrelativistic case.

Obviously, it is nowadays straightforward to develop generalized code to handle both real and complex orbitals in a single implementation without having to resort to any kind of code duplication by the use of templated code. The implementations in OpenOrbitalOptimizer accept a template data type for the orbitals as well as the orbital energies, which means that the same code can be compiled in single or double precision with real or complex orbitals, Fock, and density matrices.

### 2.1 Convergence Accelerators

The abstractions in Section 2 are a powerful tool for writing reusable code. When any algorithm is implemented following these abstractions, it should work regardless of the context it is eventually used in. Next, we will briefly review the methods that represent the state of the art in orbital optimization in the context of routine calculations. As pointed out above, it suffices to discuss the algorithms only in the context of a single symmetry block, regardless of the number of types of particles involved in the calculation, and to accumulate quantities over all symmetry blocks of all particle types. To make the discussion clearer in the following, we therefore drop the particle and symmetry block indices, and carry only the iteration index, as all the operations now refer to individual symmetry blocks of an individual type of particle.

#### 2.1.1 Pulay DIIS

We start with Pulay’s DIIS method,^19^ which was introduced to speed up the convergence of the SCF equations and is arguably still the most important technique used in electronic structure calculations today.

To devise the metric for the DIIS error, let us examine the Fock matrix in the orbital basis

10 $$F^{\mathrm{orb}}=C^{†}\mathrm{FC}$$

If the orbitals are self-consistent, that is, eq 5 applies, the Fock matrix in the orbital basis given by eq 10 is diagonal. In fact, it is easy to see that the off-diagonal elements of the Fock matrix measure orbital gradients,^3^ and that the commutator of the Fock matrix ***F*** and the density matrix ***P*** defined in eq 3

11 e=[F,P]=FP−PF

measures the orbital error, vanishing when eq 5 is satisfied.^19^ We note again here that eq 11 must be computed in the orthonormal basis,^19^ which is the reason why the choice to simplify the problem of eq 2 to eq 7 is not a limitation.

The DIIS error is given by the norm of the error vector

12 $$\epsilon^{\mathrm{DIIS}}=∥e∥$$

Common choices for this norm in various programs are the root-mean-square (rms) error

$$\epsilon^{\mathrm{DIIS}}={∥e∥}_{\mathrm{rms}}=\frac{1}{m}\sqrt{\overset{m}{\underset{i,j=1}{\sum }}{\left|e_{\mathrm{ij}}\right|}^{2}}$$ 13

as well as the *L* ^∞^ norm

$$\epsilon^{\mathrm{DIIS}}={∥e∥}_{\infty }=\underset{\mathrm{ij}}{\max}\;\left|e_{\mathrm{ij}}\right|$$ 14

By default, we use the rms norm of eq 13, which appears to be the default in many programs; however, other choices such as eq 14 are also available in OpenOrbitalOptimizer.

The idea in DIIS is to determine an optimal mixing of the Fock matrices ***F***_*i*_ of previous iterations *i*

15 $$\mathrm{F̅}=\sum_{i=1}^{m}c_{i}F_{i}$$

in order to speed up the slow convergence of the iterative Roothaan procedure. The parameter *m* in eq 15 is the length of the history. The length of the history is limited by default to *m* = 10 in OpenOrbitalOptimizer, following the default in Psi4.^54^ Note that there is a wide variation for the default value in various programs, e.g., ORCA^79^ employs *m* = 5, Q-Chem^80^ *m* = 15, and Gaussian^81^ employs *m* = 20 by default.

The DIIS extrapolation weights are determined by minimizing the extrapolated error

16 $$\mathrm{e̅}=\sum_{i=1}^{m}c_{i}e_{i}$$

with the condition that the weights sum up to one

17 $$\sum_{i=1}^{m}c_{i}=1$$

Minimization of ∥ ***e*** ∥^2^ under the constraint of eq 17 yields the Lagrangian

18 $$L=\frac{1}{2}c^{T}\mathrm{Bc}-\lambda \left(\sum_{i}c_{i}-1\right)$$

where λ is a Lagrange multiplier, and

19 $$B_{\mathrm{ij}}=\left\langle e_{i},e_{j}\right\rangle$$

are elements of the DIIS error matrix. The Lagrangian is minimized by weights that satisfy the linear system of equations^18^

20 $$\left(\begin{array}{ll} B & -1\\ -1^{T} & 0 \end{array}\right)\left(\begin{array}{l} c\\ \lambda \end{array}\right)=\left(\begin{array}{l} 0\\ -1 \end{array}\right)$$

For large history sizes, the error vectors can become linearly dependent. Hamilton and Pulay^82^ suggested multiplying the diagonal elements of eq 20 by a factor 1+*d* where *d* = 0.02 is a damping factor.

As the last row and column of eq 20 is always of the order of unity, the numerical conditioning of the linear group of equations can be improved by scaling the errors given by eq 19 such that the most recent diagonal element becomes unity.^83^ In our implementation, the matrix is normalized with respect to the smallest diagonal element *B*_*ii*_, instead; this should have no effect on the numerical behavior.

In addition, there is literature suggesting that limiting the matrices included in DIIS extrapolation may help in some cases. We use the simple criterion suggested by Chupin et al.^84^ for restarting DIIS: only those vectors *i* of the current history are included in the extrapolation that satisfy

21 $$\delta^{\mathrm{DIIS}}B_{\mathrm{ii}}\leq \min_{j}B_{\mathrm{jj}}$$

with the default parameter value δ^DIIS^ = 10^–4^.

#### 2.1.2 EDIIS and ADIIS

DIIS can be viewed as a quasi-Newton method,^84,85^ and it generally leads to fast convergence when the orbitals are close to convergence, i.e., when the orbital gradient is small. However, this also means that DIIS can only be trusted to work when one is close to a minimum; otherwise, the method can also easily converge onto a saddle point. If the initial guess is poor, other methods must therefore be used instead.

Energy DIIS^22^ (EDIIS) and augmented DIIS^23^ (ADIIS) are based on proxy energy functionals, which allow the methods to work far from the optimum. The two methods are equivalent in the case of Hartree–Fock theory,^86^ but differ in the case of density-functional theory^1,2^ (DFT) for which the energy is not a quadratic function of the density matrix.

Both EDIIS and ADIIS are defined as interpolation methods, finding an updated Fock matrix from eq 15 with the restriction of eq 17 and that the weights be non-negative

22 $$c_{i}\geq 0$$

EDIIS^22^ eponymously determines the weights *c*_*i*_ by minimizing the Hartree–Fock energy functional *E* ^HF^ for the linear combination of the density matrices

23 $$E^{\mathrm{EDIIS}}\left(c\right)=E^{\mathrm{HF}}\left(\sum_{i=0}^{k}c_{i}P_{i}\right)$$

24 $$=\sum_{i=0}^{k}c_{i}E_{i}^{\mathrm{SCF}}-\frac{1}{2}\sum_{i,j=0}^{k}c\cdot X\cdot c$$

where *E* ^SCF^ is the total energy of calculation *i*, and the matrix ***X*** is given by

25 $$X_{\mathrm{ij}}=\left\langle F_{i}-F_{j}\right|P_{i}-P_{j}\rangle$$

It is straightforward to show following the treatise of Kudin et al.^22^ that in the case of many kinds of particles, such as spin-up and spin-down electrons in spin-polarized calculations, it suffices to accumulate the ***X*** matrix over all types of particles (see proof in Appendix).

ADIIS employs the quadratic energy function of Høst et al.,^30^ which yields the expression^23^

26 $$E^{\mathrm{ADIIS}}\left(c\right)=E\left(P_{n}\right)+2\sum_{i=1}^{n}c_{i}\left\langle P_{i}-P_{n}\right|F_{n}\rangle +\sum_{i=1}^{n}\sum_{j=1}^{n}c_{i}c_{j}\left\langle P_{i}-P_{n}\right|F_{j}-F_{n}\rangle$$

The coefficients *c*_*i*_ are found by minimizing the corresponding energy, eq 24 for EDIIS and eq 26 for ADIIS, while imposing the constraints of eqs 17 and 22. Hu and Yang^23^ proposed carrying out the minimization in terms of unconstrained auxiliary variables ***x*** by implicit satisfaction of both constraints

27 $$c_{i}=\frac{x_{i}^{2}}{\sum_{j}x_{j}^{2}}$$

and such implementations appear to be widely used, e.g., in ERKALE,^50^ Psi4,^54^ PySCF,^55^ and Q-Chem.^80^ However, this is a bad idea since the minimization problem is no longer convex in ***x***. For example, we have found that this technique sometimes fails in the line search. This issue is easily understood in the case where the minimum features *c*_*i*_ = 0 for some *c*_*i*_: while the line searches will try to make the ratios of the components of ***x*** larger, the resulting ***c*** parameters are invariant to the scaling ***x*** →λ ***x***, which means that the optimization becomes slowly convergent.

Since the problem is usually rather low-dimensional, being fully determined by the length of the history, we will use a simple method inspired by Powell’s method,^87^ instead, which also appears to have been used as a fallback by Kudin et al.^22^

Both eqs 24 and 26 are quadratic forms of the type

28 $$f\left(x\right)=\frac{1}{2}x^{T}\mathrm{Ax}+b\cdot x$$

with ***A*** = ***A*** ^T^. Because the models of eq 28 are small with a dimension defined by the DIIS history length (values discussed after eq 15), it does not really matter if the algorithm used to minimize eq 28 is suboptimal, as long as it is robust, such that it always succeeds in converging (close) to the minimum of eq 28.

Mathematically, the constraints of eqs 17 and 22 correspond to optimization on the unit simplex. The initial point ***x***_0_ is found by evaluating eq 28 over the edges ***c***_*i*_, the center point ***x*** = (1/*m*,···,1/*m*)^T^, as well as slightly offset points ***x*** = (3/(*m* + 2), 1/(*m* + 2), ..., 1/(*m* + 2))^T^. The solution is then optimized starting from the point that gave the lowest value of *f*(***x***).

To ensure our solution is always in the allowed region, we update ***x*** by pairwise searches

29 $$x_{k+1}=\left(1-\lambda \right)x_{k}+\lambda c_{i}$$

where (***c***_*i*_)_*j*_ = δ_*ij*_ corresponds to an individual entry in the history. It is easy to show that the optimal value of λ for the line search of eq 29 is

30 $$\lambda =-\frac{c_{i}^{T}\mathrm{Ax}+b\cdot c_{i}}{c_{i}^{T}{\mathrm{Ac}}_{i}}$$

where the allowed region is 0 < λ ≤ 1. Searches of the form of eq 29 are repeatedly looped over *i*, until the value of *f*(***x***) does not change appreciably any more.

#### 2.1.3 Hybrid scheme of EDIIS/ADIIS followed by DIIS

Because EDIIS and ADIIS work well far from the optimum, while DIIS works well close to it, we follow Garza and Scuseria^86^ and interpolate between the two sets of weights

31 $$c^{\mathrm{hybrid}}=f^{\mathrm{int}}\left(\epsilon^{\mathrm{DIIS}}\right)c^{\mathrm{DIIS}}+\left[1-f^{\mathrm{int}}\left(\epsilon^{\mathrm{DIIS}}\right)\right]c^{\mathrm{EDIIS}/\mathrm{ADIIS}}$$

such that

- EDIIS/ADIIS is used exclusively when the DIIS error of eq 12 is large, ϵ^DIIS^ ≥ ϵ_2_ = 10^–1^,
- DIIS is used exclusively when the DIIS error is small, ϵ^DIIS^ ≤ ϵ_1_ = 10^–4^, and
- otherwise, a linear interpolation between the two is used.

Although the form eq 32 is not obvious in the equation at the bottom of page 3 of ref 86, it appears that Garza and Scuseria^86^ indeed used

32 $$f^{\mathrm{int}}\left(\epsilon^{\mathrm{DIIS}}\right)=\{\begin{array}{ll} 0, & \epsilon \geq \epsilon_{2},\\ 1-\frac{\epsilon^{\mathrm{DIIS}}-\epsilon_{2}}{\epsilon_{1}-\epsilon_{2}}, & \epsilon_{1}<\epsilon <\epsilon_{2},\\ 1, & \epsilon \leq \epsilon_{1} \end{array}$$

We note that Garza and Scuseria^86^ suggested using pure EDIIS/ADIIS if the error of the last cycle is 10% greater than the minimum error. We note that the error can increase even when the energy is lowered, and that we found this criterion to sometimes lead to convergence to local minima. For this reason, we decided not to implement this feature in OpenOrbitalOptimizer.

To choose between EDIIS and ADIIS in a given iteration of eq 31, we employ the minimal error sampling algorithm (MESA) of Garcia Chavez:^88^ we compute both the EDIIS and ADIIS interpolations, and pick the one that results in the least change to the density matrix as measured by the Frobenius norm ∥ ***P***_current_ – ***P***_new_∥_*F*_.

#### 2.1.4 History Cleanup

Even though ADIIS and EDIIS are interpolation methods that are based on solid mathematical models, using faraway points can still lead to degraded performance in DFT calculations: our exploratory calculations showed that when the orbitals undergo large changes in occupations, it is beneficial to clear the history. In our implementation, following the idea of Chupin et al.^84^ discussed above in eq 21, we keep only those density matrices in the history that satisfy

33 $$\delta^{\mathrm{history}}{∥P_{i}-P_{0}∥}_{F}\leq \min_{j\in \left[1,...,N-1\right]}{∥P_{j}-P_{0}∥}_{F}$$

where ***P***_0_ is the density matrix that led to the lowest energy, the parameter δ again has the default value δ^history^ = 10^–4^, and ∥ ∥_*F*_ denotes the Frobenius norm.

#### 2.1.5 Optimal Damping

To continue the above discussion, when a poor initial guess is used (e.g., the core Hamiltonian for a heavy atom), the orbital gradient can be huge, and the changes in the density will be large. In this case, even the ADIIS/EDIIS procedure outlined above can fail. The problem here is that some sort of restart is necessary to make ADIIS/EDIIS work with DFT methods, but when large changes in the density matrix occur, the restart may be too aggressive, cleaning up all of the preceding history.

We have solved this issue by using the optimal damping algorithm (ODA) of Cancès and Le Bris^20^ in the case of large maximum orbital gradients measured by eq 14, which is an intensive criterion, independent of system size.

In the case of a large maximum orbital gradient, the default threshold for which we set as ∥ϵ∥_∞_ ≥ 1, our procedure to relax the density matrix is as follows. In the case of a single type of particle (as in spin-restricted Hartree–Fock or DFT, for example), the relaxation problem is one-dimensional: we carry out a line search of the energy in terms of a mixture of two density matrices

34 $$E\left(\lambda \right)=E\left[\left(1-\lambda \right)P_{0}+\lambda P_{1}\right]$$

where ***P***_0_ is again the density matrix that led to the lowest energy, and ***P***_1_ is the density matrix built from the orbitals that diagonalize ***F***_0_ = ***F*** (***P***_0_). We perform a cubic interpolation to find λ in eq 34.^89^

If *E*(***P***_1_) < *E*(***P***_0_), we accept the step. Otherwise, we already know *E*(0) = *E*(***P***_0_) = *E*_0_ and ***F***_0_ = ***F*** (***P***_0_), as well as the values at λ = 1, *E*(1) = *E*(***P***_1_) = *E*_1_ and ***F***_1_ = ***F*** (***P***_1_). The derivatives with respect to λ are given by these data as *E*′(0) = Tr ***F***_0_(***P***_1_–***P***_0_) and *E*′(1) = Tr ***F***_1_(***P***_1_–***P***_0_). The four data points *E*(0), *E*′(0), *E*(1) and *E*(1) afford a cubic fit.

If the cubic fit predicts a minimum at 0 < λ* < 1 (if there are two minima in the allowed region we pick the lower one predicted by the fit), we compute the corresponding natural orbitals and orbital occupations

35 $$\left(1-\lambda^{*}\right)P_{0}+\lambda^{*}P_{1}={\mathrm{CfC}}^{†}$$

and evaluate the corresponding total energy and Fock matrix.

If there are several types of particles, the optimization problem is many-dimensional. For example, for the case of spin-unrestricted Hartree–Fock or density-functional theory, we have to optimize

$$E\left(\lambda_{\alpha },\lambda_{\beta }\right)=E\left[P^{\alpha }\left(\lambda_{\alpha }\right),P^{\beta }\left(\lambda_{\beta }\right)\right]$$ 36

in the unit square **λ** = (λ_α_, λ_β_) ∈ [0,1]^2^/(0, 0), with separate interpolations of the form eq 35 for either spin. Knowing the current Fock matrices ***F***_0_^σ^ for both spins, we can build the new density matrices ***P***_1_^σ^, and compute the energy gradients with respect to the mixing factors

37 $$\partial E/\partial \lambda_{\sigma }=\mathrm{Tr}\;F_{0}^{\sigma }\left(P_{1}^{\sigma }-P_{0}^{\sigma }\right)$$

The negative gradient −∇_**λ**_ *E* then determines the search direction. Since the search always starts from the current solution, which corresponds to **λ** = **0**, the components of the search direction ***s*** are obtained as

38 $$s_{i}=\max\left(0,-\partial E/\partial \lambda_{i}\right)$$

to ensure we are in the allowed region. The trial **λ** ^trial^ is then obtained by stepping to the boundary,

39 $$\lambda^{\mathrm{trial}}=\frac{s}{\max_{i}s_{i}}$$

The energy *E*(**λ** ^tr^) is then evaluated, and again if *E*(**λ** ^tr^) < *E*(**0**) the step is accepted. Otherwise, we perform a cubic interpolation along **λ** ^tr^ exactly as in the case of one dimension. Note that even though the example discussed herein only had two types of particles, this procedure is valid for an arbitrary number of types of particles.

ODA steps are repeated until the maximum orbital gradient becomes acceptable. The history generated by the ODA algorithm may then be used by the ADIIS/EDIIS interpolation and DIIS extrapolation methods.

## 3 Design Choices

Arguably the most important design choice in any project is the license. In order to promote the reusability of the library and avoid the need for duplicate solutions,^36^ we chose to license OpenOrbitalOptimizer under the Mozilla Public License that is also used in Libxc,^90^ which is nowadays used by over 40 programs including also several commercial programs.

We chose to make OpenOrbitalOptimizer a header-only library in order to facilitate its inclusion in various programs. The language was chosen as C++ as the standard in scientific computing; there are many recent quantum chemistry codes in C++, and the C++ language makes templating easy.

Moreover, as the main tasks of the library have to do with linear algebra, the library is developed on top of the Armadillo library,^91^ which offers a convenient syntax for matrix and vector operations, as well as the required support for templated datatypes that are used throughout OpenOrbitalOptimizer. The use of Armadillo within the library does not restrict the use of the library in other codes, since the matrix data is easy to convert to other formats in the library interface.

The main design decision in OpenOrbitalOptimizer is the limitation to problem sizes that are most common in quantum chemistry, that is, matrix sizes that can be stored in core memory, and diagonalized in full. This is a conscious design decision, motivated by the want to have a lean library that is easy to work with, and is easy to include in other codes. The library can still be potentially useful in other contexts, such as calculations in plane-wave basis sets, as one can always find a smaller orbital basis to use OpenOrbitalOptimizer in, for example by computing the *N* lowest crystalline or molecular orbitals for a guess Hamiltonian by iterative diagonalization.

Since evaluation of the Fock matrix can be more efficient in terms of orbital coefficients and occupation numbers rather than a density matrix (this is the case with resolution-of-the-identity techniques for exact exchange, as well as DFT in large orbital basis sets, for example), the basic data that OpenOrbitalOptimizer works with are the molecular orbital coefficients ***C*** and occupation numbers ***f*** for all symmetry blocks.

The library is used by defining the numbers of blocks per particle type (which also implicitly defines the number of particle types), the maximum occupation in each block, the number of particles of each type, a function to evaluate the Fock matrix and total energy from the given ***C*** and ***f***, and the descriptions of each of the blocks. Calculations can be initialized either by supplying initial guesses for ***C*** and ***f***, or by supplying a guess for the Fock matrix ***F***.

In our experience, the SCF implementations in many programs can converge onto a higher energy than what was encountered during the SCF iterations. A key goal in our implementation is to avoid this by never erasing the lowest-energy estimate for the solution. We always keep the lowest-energy solution first in our stack, after which the iterates are sorted in decreasing iteration number. When the stack is full, we erase the oldest nonminimal solution, which is kept at the end of the stack.

This procedure has the potential pitfall in that it can get stuck. We decided to resolve this issue by triggering an alternative strategy in the case *N*/2 consecutive (A/E)­DIIS iterations have failed to result in a decrease of the total energy, *N* being the maximum allowable history length: our tit-for-tat strategy is to carry out the next *N*/2 iterations with ODA in the hopes to make sure that the solution gets sufficiently far away from the stagnant region for (A/E)­DIIS to recover its usefulness.

## 4 Computational Details

Although OpenOrbitalOptimizer has already been interfaced with several programs, such as HelFEM^51,52,92^ and Psi4,^54^ we exemplify the library with nuclear-electronic orbital^62–64^ (NEO) Hartree–Fock (HF) calculations that we implemented in a plugin to the ERKALE program.^50,93^

NEO-HF consists of solving a coupled set of equations for the protons and electrons, which read in the atomic-orbital basis as^62^

40 $$\{\begin{array}{l} F^{e}C^{e}=S^{e}C^{e}P^{e},\\ F^{p}C^{p}=S^{p}C^{p}P^{p} \end{array}$$

The electronic and protonic Fock matrices are given by

41 $$F^{e}=T^{e}+V^{e}+J^{\mathrm{ee}}+K^{\mathrm{ee}}+J^{\mathrm{pe}}$$

42 $$F^{p}=T^{p}+V^{p}+J^{\mathrm{pp}}+K^{\mathrm{pp}}+J^{\mathrm{ep}}$$

where ***T*** ^e^ is the electronic kinetic energy matrix, ***V*** ^e^ are the nuclear attraction integrals for the Born–Oppenheimer nuclei, ***J*** ^ee^ is the electron–electron Coulomb matrix, ***K*** ^ee^ is the electron–electron exchange matrix, and ***J*** ^pe^ is the proton–electron Coulomb matrix, i.e., the Coulomb potential that the protons generate for the electrons. As usual in NEO, the protons are assumed to be in a high-spin state in all calculations.

As electronic basis sets, we consider the polarization consistent pc-n and aug-pc-n basis sets of Jensen.^94,95^ Following our recent work in ref 96, we uncontract the electronic basis set on the quantum protons, as this leads to significant improvements in the precision of the calculation; this procedure is denoted as uncHq-. The protonic basis set is PB4-F1 ^97^ in all calculations. All basis sets are taken from the Basis Set Exchange.^98^

We use density fitting in all calculations with autogenerated auxiliary basis sets. As recently reviewed by Pedersen et al.,^99^ there is a close connection between density fitting and Cholesky decompositions. Liu et al.^100^ showed that the Cholesky decomposition yields good results in NEO calculations when the decomposition is carried out for the union of the protonic and electronic basis functions; we thus generated the auxiliary basis set from pivoted Cholesky decompositions for the union of the protonic and electronic basis functions following our recent work.^101,102^ We then used the same auxiliary basis set for both the electrons and the protons.

We assess the accuracy of our density-fitted NEO-HF implementation against calculations performed with exact integrals in Q-Chem.^80^ The two-electron integral threshold was tightened to ϵ = 10^–12^ in Q-Chem. The basis set linear dependence thresholds were set to 10^–5^ in both codes. The Coulomb overlap matrix in the density fitting approach was inverted using the pivoted Cholesky technique of ref 14, using a 10^–8^ threshold to choose the pivot functions, and a 10^–7^ linear dependence threshold for the canonical orthonormalization step.

Solving eq 40 requires simultaneous initial guesses for both the electrons and the protons. A reasonable guess for the electrons is obtained from standard atomic guesses, as proton delocalization effects are likely localized in the electronic structure; we used a superposition of atomic potentials^10,11^ for the present calculations. However, we found that guessing the protonic Fock matrix based on the electronic solution leads to poor results.

Instead, we found that a minimal 1s protonic basis guess with exponent 16 √2 ≈ 22.627 adopted from the 8s8p8d even-tempered basis of Yang et al.^103^ leads to qualitatively correct electronic and protonic orbitals, as calculations in the larger basis sets with this read-in guess converge to the DIIS region in just a few iterations. Performing this initial calculation has practically no overhead, since there are no protonic degrees of freedom to optimize in the minimal basis.

We note that Liu et al.^65^ described using a guess where the proton occupies the tightest protonic basis function for each quantum nucleus—which we assume to refer to an s function. Our guess is thus somewhat similar to that of Liu et al., but our guess is independent of the protonic basis used in the actual calculation. Note that the tightest basis function in the protonic 8s8p8d basis of Yang et al.^103^ has an exponent of 32.0, while the tightest s function in PB4-F1 ^97^ has an exponent 28.950.

Highlighting the robustness of the reusable SCF solver in OpenOrbitalOptimizer, NEO-HF was implemented in both the simultaneous and stepwise fashions. In the stepwise approach, each macroiteration consists of first solving the electronic problem at fixed proton–electron Coulomb potential, then updating the electron–proton Coulomb potential, and then solving the protonic problem in this fixed external Coulomb potential. The macroiteration terminates by the update of the proton–electron Coulomb potential. The energy is evaluated in full at every iteration by adding in the quantum mechanical energy for the fixed particles (protons for electrons, and electrons for protons).

Due to the differences in Fock matrix constructions, the simultaneous and stepwise approaches can have very different costs per iteration, as illustrated by Table 1. Even though the simultaneous optimization has been found to converge faster,^65^ the electronic basis set is much larger than the protonic one in typical calculations; this is especially the case when one is interested in going toward the complete basis set limit, see Khan and Tonner-Zech^104^ and our recent work in ref 96. Even though the protonic problem tends to be more difficult to converge, requiring many more iterations than the electronic one, the protonic iterations are usually extremely cheap in comparison to the electronic ones.

**Table 1:** Terms to Calculate in Every Iteration in NEO-HF^a^

matrices	stepwise electronic	stepwise protonic	simultaneous
***J***^ee^, ***K***^ee^	yes	no	yes
***J***^pp^, ***K***^pp^	no	yes	yes
***J*** ^ep^	after convergence	no	yes
***J*** ^pe^	no	after convergence	yes

a Each step of the electronic (protonic) problem only requires computing the corresponding exchange and Coulomb matrices, while the electron-proton (proton-electron) Coulomb term is only evaluated at convergence.

We found the speed of convergence to be greatly influenced by the DIIS history length in exploratory calculations. A history length of 20 matrices was used in all the calculations reported herein; going higher did not appear to lead to improved convergence.

## 5 Results

We consider NEO-HF calculations on the protonated water tetramers H_3_O^+^(H_2_O)_3_ with the four geometries used by Liu et al.,^65^ which were originally determined by Heindel et al.^105^ The geometries correspond to ring, eigen, ciszundel, and transzundel structures. As all nine protons were modeled quantum mechanically, these calculations are a good check for the robustness of the convergence accelerators. All calculations were converged to a root-mean-square (rms) orbital gradient, i.e., DIIS error of 10^–7^.

Calculations were carried out in both the simultaneous and stepwise procedures. The stepwise procedure was carried out until the energy changed less than 10^–7^ E_h_ in a macroiteration, at which point the calculations switched to the simultaneous algorithm; in contrast, calculations using the simultaneous approach employ that algorithm from the start.

Calculations were furthermore performed with auxiliary basis sets of three sizes: the “small”, “large”, and “verylarge” auxiliary basis sets generated with the procedure of ref 102. As previous experience shows that at least a triple-ζ orbital basis is required for generating reliable auxiliary basis sets,^101^ data presented for the double-ζ pc-1 and aug-pc-1 orbital basis sets employ the auxiliary basis set of the corresponding triple-ζ orbital basis set.

The number of Fock matrix builds required in the various calculations is shown in Table 2. As the table demonstrates, the total number of Fock matrix builds is indeed smaller in the simultaneous mode, as Liu et al.^65^ found. However, the stepwise solution requires roughly a third fewer electronic Fock matrix builds than the simultaneous solution; the electron–proton and proton–electron Coulomb matrices also need to be built a considerably smaller number of times. Even though the stepwise solution requires 2–3 times more protonic Fock matrices, the protonic basis is typically a small fraction of the size of the electronic basis, and these builds are extremely cheap.

**Table 2:** Number of Fock Matrix Builds Needed to Converge the NEO-HF Calculations with the “large” Auxiliary Basis for Various Geometries of the Protonated Water Tetramer: *N*_sim_ is the Number of Fock Builds in the Simultaneous Electronic-Protonic SCF, while *N*_elec_ and *N*_prot_ are the Total Numbers of Electronic and Protonic Fock Builds in the Stepwise SCF Procedure (*c.f.* Table 1)­^a^

basis	geometry	*N*_sim_	*N*_elec_	*N*_prot_	*N*_ep/*pe*_	Δ*E*
uncHq-pc-**1**	ring	124	93	298	32	–4.6 × 10^–8^
uncHq-pc-**1**	eigen	123	91	276	35	–7.2 × 10^–8^
uncHq-pc-**1**	ciszundel	127	91	281	34	–7.7 × 10^–8^
uncHq-pc-**1**	transzundel	126	92	285	35	–8.6 × 10^–8^
uncHq-**aug**-pc-**1**	ring	108	69	229	21	–2.8 × 10^–8^
uncHq-**aug**-pc-**1**	eigen	100	60	210	12	–1.0 × 10^–8^
uncHq-**aug**-pc-**1**	ciszundel	99	65	205	20	–3.4 × 10^–8^
uncHq-**aug**-pc-**1**	transzundel	99	65	206	20	–3.4 × 10^–8^
uncHq-pc-**2**	ring	134	105	342	44	–1.0 × 10^–7^
uncHq-pc-**2**	eigen	132	105	329	47	–1.3 × 10^–7^
uncHq-pc-**2**	ciszundel	141	96	339	35	–1.1 × 10^–7^
uncHq-pc-**2**	transzundel	132	104	346	43	–9.7 × 10^–8^
uncHq-**aug**-pc-**2**	ring	113	69	292	17	–7.3 × 10^–8^
uncHq-**aug**-pc-**2**	eigen	111	68	290	16	–6.3 × 10^–8^
uncHq-**aug**-pc-**2**	ciszundel	114	69	299	17	–5.5 × 10^–8^
uncHq-**aug**-pc-**2**	transzundel	112	70	294	17	–7.3 × 10^–8^
uncHq-pc-**3**	ring	424	211	946	54	–3.0 × 10^–7^
uncHq-pc-**3**	eigen	532	183	836	48	2.0 × 10^–7^
uncHq-pc-**3**	ciszundel	476	211	966	55	–1.0 × 10^–6^
uncHq-pc-**3**	transzundel	361	188	831	48	–5.9 × 10^–8^
uncHq-**aug**-pc-**3**	ring	379	201	942	54	2.7 × 10^–7^
uncHq-**aug**-pc-**3**	eigen	444	173	837	48	1.8 × 10^–6^
uncHq-**aug**-pc-**3**	ciszundel	510	195	950	54	–6.7 × 10^–8^
uncHq-**aug**-pc-**3**	transzundel	282	179	842	49	1.3 × 10^–6^

a *N*_ep/pe_ shows the number of times the electron-proton and proton-electron coupling terms were calculated, while Δ*E* = *E*(simultaneous) – *E*(separate) shows the difference in the final total energies.

Furthermore, we find that the simultaneous solution slows down considerably when going to larger electronic basis sets, and that the contrast to the stepwise algorithm becomes even larger.

We assess the precision of the calculations with the three sizes of auxiliary basis sets in Table 3. In the larger orbital basis sets, the fit errors in the total NEO-HF energies are just a few μE_h_, causing no concern about the accuracy of the calculations. The fitting errors in the total energy are slightly larger in the calculations with the smallest orbital basis sets, but the error of 9 × 10^–5^E_h_ ≈ 0.06 kcal/mol is negligible. Note also that the error is very systematic across the geometries, and will therefore exhibit systematic error cancellation in computing isomer or reaction energies, for instance.

**Table 3:** Precision of the RI-NEO-HF Calculations of This Work (TW), Compared to Analogous Calculations Carried Out with Traditional Two-Electron, Two-Proton, Electron-Proton, and Proton-Electron Integrals with Q-Chem

^a^basis	model	eigen	transzundel	ciszundel	ring
uncHq-pc-**1** ^b^	Q-Chem	–304.0829921	–304.0763508	–304.0760041	–304.0739284
uncHq-pc-**1** ^b^	TW, “small”	0.0000922	0.0000922	0.0000922	0.0000939
uncHq-pc-**1** ^b^	TW, “large”	0.0000561	0.0000560	0.0000560	0.0000574
uncHq-pc-**1** ^b^	TW, “verylarge”	0.0000207	0.0000219	0.0000221	0.0000239
uncHq-pc-**2**	Q-Chem	–304.2805872	–304.2746806	–304.2743526	–304.2705210
uncHq-pc-**2**	TW, “small”	0.0000128	0.0000129	0.0000128	0.0000152
uncHq-pc-**2**	TW, “large”	0.0000010	0.0000010	0.0000010	0.0000020
uncHq-pc-**2**	TW, “verylarge”	–0.0000030	–0.0000016	–0.0000015	–0.0000006
uncHq-pc-**3**	Q-Chem	–304.3062376	–304.3003964	–304.3001021	–304.2963777
uncHq-pc-**3**	TW, “small”	0.0000039	0.0000031	0.0000027	0.0000033
uncHq-pc-**3**	TW, “large”	0.0000007	0.0000004	–0.0000001	0.0000007
uncHq-pc-**3**	TW, “verylarge”	–0.0000002	–0.0000010	–0.0000008	–0.0000003
uncHq-**aug**-pc-**1** ^b^	Q-Chem	–304.0967476	–304.0902633	–304.0904477	–304.0896750
uncHq-**aug**-pc-**1** ^b^	TW, “small”	0.0000628	0.0000629	0.0000628	0.0000628
uncHq-**aug**-pc-**1** ^b^	TW, “large”	0.0000232	0.0000225	0.0000229	0.0000220
uncHq-**aug**-pc-**1** ^b^	TW, “verylarge”	0.0000223	0.0000222	0.0000220	0.0000204
uncHq-**aug**-pc-**2**	Q-Chem	–304.2820253	–304.2761474	–304.2758457	–304.2721887
uncHq-**aug**-pc-**2**	TW, “small”	0.0000046	0.0000047	0.0000048	0.0000051
uncHq-**aug**-pc-**2**	TW, “large”	–0.0000016	–0.0000027	–0.0000027	–0.0000027
uncHq-**aug**-pc-**2**	TW, “verylarge”	–0.0000021	–0.0000023	–0.0000025	–0.0000039
uncHq-**aug**-pc-**3**	Q-Chem	–304.3064263	–304.3005800	–304.3002842	–304.2965622
uncHq-**aug**-pc-**3**	TW, “small”	0.0000016	0.0000008	0.0000003	0.0000004
uncHq-**aug**-pc-**3**	TW, “large”	–0.0000004	0.0000003	0.0000003	0.0000007
uncHq-**aug**-pc-**3**	TW, “verylarge”	–0.0000022	–0.0000009	–0.0000034	–0.0000014

a The data are Shown as deviations from the Q-chem total energy. All values are in *E*_h_.

b Calculation employed auxiliary basis set generated for the corresponding triple-ζ basis: uncHq-pc-2 or uncHq-aug-pc-2.

Clear improvement in precision can be observed for the smaller orbital basis sets when using larger and larger auxiliary basis sets, but this pattern is not so evident in the largest calculations, which we interpret as an issue with finite numerical precision, as the large auxiliary basis sets start to exhibit linear dependencies.

## 6 Summary and Conclusions

We have presented OpenOrbitalOptimizer,^45^ a reusable open-source header-only templated C++ library for orbital optimization and self-consistent field (SCF) calculations. OpenOrbitalOptimizer aims to either fully eliminate existing disparate SCF implementations in various programs, or alternatively to supplement their functionalities by offering access to consistent implementations of various SCF methodologies. Attesting to the power of modern programming paradigms, the present implementation is only around 2400 lines of C++ code, including extensive comments.

OpenOrbitalOptimizer offers a unified interface for solving coupled SCF equations of an arbitrary number of types of particles, with an arbitrary number of symmetries. OpenOrbitalOptimizer has already been interfaced with HelFEM,^51–53,57,60,92^ ERKALE,^50,93^ and Psi4,^54^ and we hope to see many more interfaces with other programs. While none of the currently interfaced codes perform fully relativistic or solid-state calculations, the necessary extensions discussed in the Methods section should be straightforward to make in OpenOrbitalOptimizer.

OpenOrbitalOptimizer is openly available free of charge under the Mozilla Public License, with the main project hosted at https://github.com/susilehtola/OpenOrbitalOptimizer. It runs on Linux, MacOS, and Windows.

The development of OpenOrbitalOptimizer was greatly motivated by the needs to support nuclear-electronic orbital (NEO) calculations,^62–64^ which feature also quantum protons in addition to electrons. We exemplified OpenOrbitalOptimizer with NEO Hartree–Fock calculations on protonated water clusters, and described a simple but powerful initial guess for the protons. Calculations were carried out both with simultaneous electron–proton SCF, as well as the stepwise SCF procedure consisting of alternating SCF procedures for the electrons and protons, respectively. We found the stepwise SCF procedure to exhibit stabler convergence.

In future work, we aim to extend the functionality of OpenOrbitalOptimizer for calculations on systems with degeneracies at the Fermi level, in which case the Aufbau theorem does not hold and the occupations of the degenerate orbitals must instead be explicitly optimized. Although our main interest in this problem arises from atomic calculations,^10,53,57,59,106^ degenerate occupations can sometimes be also found in molecules,^107–109^ and are likely the main challenge in large-scale black-box applications of DFT. A reusable implementation of such an approach in OpenOrbitalOptimizer would then allow exploring fractional-occupation solutions in any context, given the flexibility of the present approach.

One other potential avenue for future development are interfaces to other languages, such as Python and Fortran, which is easiest to accomplish via a C language interface.^36^ We hope to be able to accommodate a C language interface in the future. While the C++ library is header-only, the C interface will result in a compiled binary, which may complicate software distribution.

## Appendix: Generalization of EDIIS to Many Kinds of Particles

Let us consider the extension of EDIIS^22^ to the case where the energy depends on density matrices {***P***_σ_} of several types of particles σ, which may be represented using different basis sets. The Hartree–Fock energy functional, which is used to build the EDIIS proxy energy, can be summarized as

$$E^{\mathrm{HF}}\left(\{P_{\sigma }\}\right)=\underset{\sigma }{\sum }\mathrm{Tr}\;h_{\sigma }P_{\sigma }+\frac{1}{2}\underset{\sigma }{\sum }\mathrm{Tr}\;G_{\sigma }\left(P_{\sigma }\right)P_{\sigma }+\underset{\sigma^{\prime }\neq \sigma }{\sum }\mathrm{Tr}\;J_{\sigma }\left(P_{\sigma \prime }\right)P_{\sigma }$$
43

where ***h***_σ_ is the one-electron Hamiltonian, ***G***_σ_(***P***_σ_) contains the Coulomb and exchange contributions, and ***J*** is just the Coulomb interaction, which satisfies Tr ***J***_σ′_(***P***_σ_) ***P***_σ′_ = Tr ***J***_σ_(***P***_σ′_) ***P***_σ_ but we include both terms to make the end result symmetric by construction. The Fock matrix corresponding to eq 43 is

44
$$F_{\sigma }=\partial E^{\mathrm{HF}}/\partial P_{\sigma }=h_{\sigma }+G_{\sigma }\left(P_{\sigma }\right)+J_{\sigma }\left(P_{\sigma \prime }\right)$$

and thereby the difference between the Fock matrices of iterations *j* and *i* is

45
$$F_{\sigma }^{\left(j\right)}-F_{\sigma }^{\left(i\right)}=G_{\sigma }\left(P_{\sigma }^{\left(j\right)}\right)-G_{\sigma }\left(P_{\sigma }^{\left(i\right)}\right)+J_{\sigma }\left(P_{\sigma \prime }^{\left(j\right)}\right)-J_{\sigma }\left(P_{\sigma \prime }^{\left(i\right)}\right)$$

In the proof, one merely extends the definition of the EDIIS energy functional to the case of different types of particles, while still employing the same extrapolation coefficients *c*_*i*_ ≥ 0 with 1 = ∑_*i*_ *c*_*i*_:

46
$$f^{\mathrm{EDIIS}}\left(c\right)=E^{\mathrm{HF}}\left(\left\{\sum_{i}c_{i}P_{\sigma }^{\left(i\right)}\right\}\right)$$

As noted by Kudin et al.,^22^ the manipulation is straightforward; it suffices to note that one first needs to insert 1 = ∑_*j*_ *c*_*j*_ to arrive at an expression which looks like ∑_*ij*_ *A*_*ij*_ *P*_*i*_, where ***A*** is an antisymmetric quantity, *A*_*ij*_ = – A_*ji*_. In the second step, splitting the sum in two copies and interchanging the indices *i* ↔ *j* in the other term, one gets $\sum_{\mathrm{ij}}A_{\mathrm{ij}}P_{i}=\frac{1}{2}\sum_{\mathrm{ij}}A_{\mathrm{ij}}P_{i}$ + $\frac{1}{2}\sum_{\mathrm{ij}}A_{\mathrm{ji}}P_{j}$ = $\frac{1}{2}\sum_{\mathrm{ij}}A_{\mathrm{ij}}\left(P_{i}-P_{j}\right)$ . These two steps are sufficient to manipulate eq 46 into the form

47
$$f^{\mathrm{EDIIS}}\left(c\right)=\sum_{i}c_{i}E^{\mathrm{UHF}}\left(\{P_{\sigma }^{\left(i\right)}\}\right)-\frac{1}{4}\sum_{\mathrm{ij}}c_{i}c_{j}\sum_{\sigma }\mathrm{Tr}\left[F_{\sigma }\left(P_{\sigma }^{\left(j\right)}\right)-F_{\sigma }\left(P_{\sigma }^{\left(i\right)}\right)\right]\times \left(P_{\sigma }^{\left(j\right)}-P_{\sigma }^{\left(i\right)}\right)$$

which remarkably has the same form as eq 46, where now the quadratic term just has a sum over the particle types. This also proves that EDIIS works more generally, for example with the nuclear-electronic orbital method, as it suffices to sum over the contributions over the various particle types. As EDIIS and ADIIS coincide in the case of Hartree–Fock, the same observation should also hold for ADIIS.
